# Author Correction: Blunting neuroinflammation with resolvin D1 prevents early pathology in a rat model of Parkinson’s disease

**DOI:** 10.1038/s41467-019-12538-2

**Published:** 2019-10-14

**Authors:** Paraskevi Krashia, Alberto Cordella, Annalisa Nobili, Livia La Barbera, Mauro Federici, Alessandro Leuti, Federica Campanelli, Giuseppina Natale, Gioia Marino, Valeria Calabrese, Francescangelo Vedele, Veronica Ghiglieri, Barbara Picconi, Giulia Di Lazzaro, Tommaso Schirinzi, Giulia Sancesario, Nicolas Casadei, Olaf Riess, Sergio Bernardini, Antonio Pisani, Paolo Calabresi, Maria Teresa Viscomi, Charles Nicholas Serhan, Valerio Chiurchiù, Marcello D’Amelio, Nicola Biagio Mercuri

**Affiliations:** 1 Department of Experimental Neurosciences, IRCCS Santa Lucia Foundation, 00143 Rome, Italy; 2 Department of Medicine and Department of Science and Technology for Humans and Environment, University Campus Bio-medico, 00128 Rome, Italy; 3 Department of Systems Medicine, University of Rome ‘Tor Vergata’, 00133 Rome, Italy; 4 Department of Philosophy, Human, Social and Educational Sciences, University of Perugia, 06123 Perugia, Italy; 5 Department of Clinical and Behavioural Neurology, IRCCS Santa Lucia Foundation, 00143 Rome, Italy; 6 Institute of Medical Genetics and Applied Genomics, University of Tübingen, 72076 Tübingen, Germany; 7 Department of Experimental Medicine and Surgery, University of Rome ‘Tor Vergata’, 00133 Rome, Italy; 8 Neurology Clinic, Department of Medicine, University of Perugia, Santa Maria della Misericordia Hospital, 06156 Perugia, Italy; 9 Institute of Histology and Embryology, Università Cattolica del Sacro Cuore, 00168 Rome, Italy; 10 Center for Experimental Therapeutics and Reperfusion Injury, Department of Anaesthesiology, Perioperative and Pain Medicine, Brigham and Women’s Hospital and Harvard Medical School, 02115 Boston, MA USA

**Keywords:** Neuroimmunology, Parkinson's disease

Correction to: *Nature Communications* 10.1038/s41467-019-11928-w, published online 02 September 2019.

The original version of this Article contained an error in Fig. 2d. The data points in Fig. 2d were inadvertently duplicated from Fig. 2a. This has now been corrected in both the PDF and HTML versions of the Article. The Source Data corresponding to this panel and published with the article was correct and has not been updated.

