# Supplementary material for: Blunting neuroinflammation with resolvin D1 prevents early pathology in a rat model of Parkinson’s disease
Source: Nat Commun. 2019 Sep 2;10:3945. doi: 10.1038/s41467-019-11928-w (PMC6718379; doi:10.1038/s41467-019-11928-w)
Supplement: Supplementary file 1 — Supplementary Information [file 41467_2019_11928_MOESM1_ESM.pdf]

# Blunting neuroinflammation with resolvin D1 prevents early pathology in a rat model of Parkinson’s disease

Krashia et al.

## SUPPLEMENTARY INFORMATION

Supplementary Figures 1-8

Supplementary Table 1

SUPPLEMENTARY FIGURES:

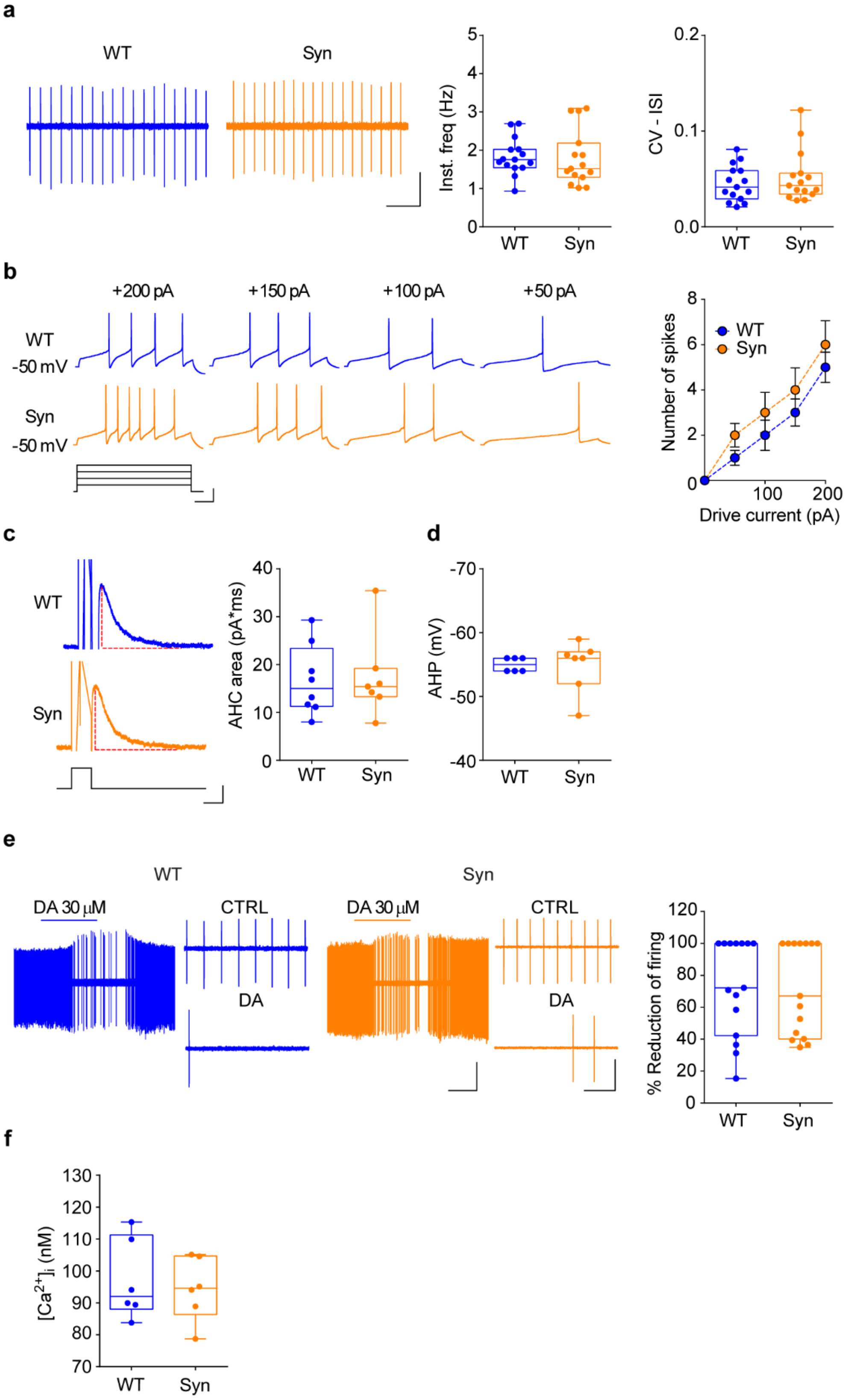


**Supplementary Figure 1: Electrophysiological properties and $Ca^{2+}$ levels in SNpc DA neurons of 2-month-old rats.** (**a**) Spontaneous firing patterns from 2-month-old DA neurons, showing similar

regular firing across genotypes (scale: 2 s, 0.2 mV). The plots indicate the firing rate and coefficient of variation of the interspike interval (15 neurons, 3 animals each; frequency: Welch's t-test P=0.860; CV-ISI: Mann-Whitney test P=0.595). (**b**) Example of current-clamp recordings (scale: 100 ms; 20 mV, 100 pA) and mean AP plot (± s.e.m) in response to depolarising current pulses (6 WT, 7 Syn neurons, 3 rats each; two-way repeated-measures ANOVA: genotype×drive current, $F_{4,44}$=0.89, P=0.478; current, $F_{4,44}$=45.87, P<1.00x$10^{-4}$; genotype, $F_{1,11}$=0.829, P=0.382). (**c**) Traces of AHC in DA neurons after a voltage step to 0 mV (scale: 100 ms; 50 pA, 50 mV). The plot shows the area under the traces (indicated by the dashed line). No differences were detected between genotypes (8 WT, 7 Syn cells, 3 rats each; 2-tailed Welch's t-test P=0.884). (**d**) After-hyperpolarisation potential (AHP) during the first evoked AP (6 WT, 7 Syn neurons, 3 rats each; 2-tailed Mann-Whitney test P=0.427). (**e**) Extracellular firing response to bath-applied DA (30 µM) for 2 min (scale: 1 min, 0.2 mV). Expanded traces (scale: 1 s) show firing in control conditions (CTRL) and during DA. The plot shows the % of DA-induced inhibition (15 neurons, 3 animals each; P=0.908 with Mann-Whitney test). (**f**) Somatic cytoplasmic [$Ca^{2+}$] in DA neurons at -60 mV (6 cells, 3 rats each; P=0.694 with Welch's t-test). Source data are provided as a Source Data file.

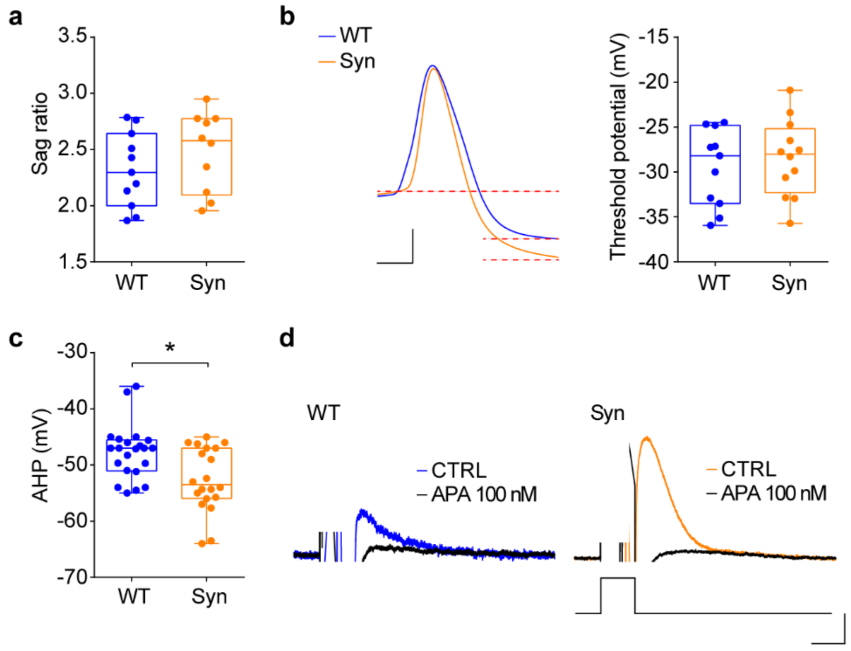


**Supplementary Figure 2: Additional properties of SNpc DA neurons in 4-month-old rats.** (**a**) Sag ratio, measured as the ratio of the steady-state versus peak potential during sub-threshold responses to -200 pA current injections (11 WT, 10 Syn neurons, 3 rats each; P=0.286 with 2-tailed Welch's t-test). (**b,c**) Superimposed APs patterns (scale: 1 ms, 15 mV). The dashed lines represent the threshold and the negative after-hyperpolarisation peaks. The plots show similar threshold potential (**b**; 11 WT and 12 Syn neurons, 5 rats each; P=0.570 with 2-tailed Welch's t-test), but more negative after-hyperpolarisation potential (AHP; **c**) in DA cells from 4-month-old Syn rats (22 WT, 20 Syn neurons, 5 rats each; AHP: *P=0.012 with 2-tailed Mann-Whitney test). (**d**) AHC in response to a voltage step from -60 to 0 mV in aCSF or after bath application of 100 nm apamin, an SK channels inhibitor (black traces; scale: 100 ms; 50 pA, 50 mV).

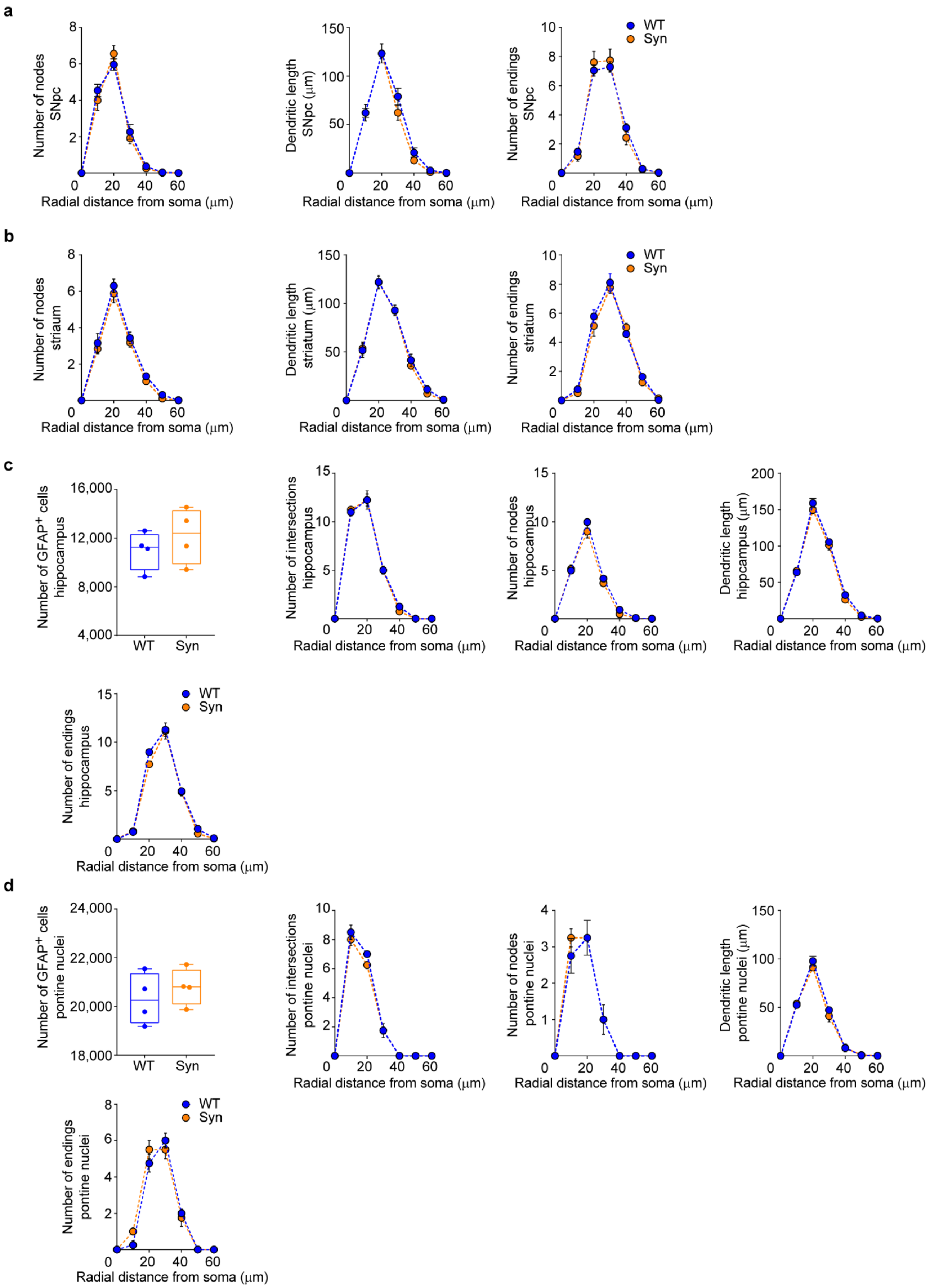
a
Number of nodes SNpc
Dendritic length SNpc (μm)
Number of endings SNpc
WT
Syn
Radial distance from soma (μm)
b
Number of nodes striaum
Dendritic length striatum (μm)
Number of endings striatum
c
Number of GFAP+ cells hippocampus
Number of intersections hippocampus
Number of nodes hippocampus
Dendritic length hippcampus (μm)
Number of endings hippocampus
d
Number of GFAP+ cells pontine nuclei
Number of intersections pontine nuclei
Number of nodes pontine nuclei
Dendritic length pontine nuclei (μm)
Number of endings pontine nuclei

**Supplementary Figure 3: Analysis of GFAP$^+$ astrocytes in 4-month-old rats.** (**a**) Morphological parameters in the SNpc (4 WT, 4 Syn rats; nodes: two-way repeated-measures ANOVA for genotype×radial distance, $F_{6,36}$=1.02, P=0.426; genotype, $F_{1,6}$=0.108, P=0.753; distance, $F_{6,36}$=200.3, P<1.00x10$^{-4}$; 0-60 μm: P>0.05 with Bonferroni's; dendritic length: genotype×radial distance $F_{6,36}$=1.02, P=0.427; genotype, $F_{1,6}$=0.828, P=0.398; distance, $F_{6,36}$=225.8, P<1.00x10$^{-4}$; WT-Syn for 0-60 μm: P>0.05 with Bonferroni's; number of endings: genotype×distance $F_{6,36}$=0.702, P=0.650; genotype, $F_{1,6}$=3.00x10$^{-4}$, P=0.986; distance, $F_{6,36}$=173.2, P<1.00 x 10$^{-4}$; 0-60 μm: P>0.05 with Bonferroni's test). (**b**) As in (**a**) but for astrocytes in dorsolateral striatum (4 WT, 4 Syn rats; nodes; two-way repeated-measures ANOVA for genotype×distance, $F_{6,36}$=0.217, P=0.969; genotype, $F_{1,6}$=1.04, P=0.348; distance, $F_{6,36}$=171.5, P<1.00x10$^{-4}$; 0-60 μm: P>0.05 with Bonferroni's; dendritic length: genotype×radial distance $F_{6,36}$=0.225, P=0.966; genotype, $F_{1,6}$=0.11, P=0.751; distance, $F_{6,36}$=284.3, P<1.00 x 10$^{-4}$; WT-Syn for 0-60 μm: P>0.05 with Bonferroni's; endings: genotype×distance $F_{6,36}$=0.890, P=0.513; genotype, $F_{1,6}$=0.335, P=0.584; distance, $F_{6,36}$ = 255.2, P<1.00x10$^{-4}$; 0-60 μm: P>0.05 with Bonferroni's). (**c**) GFAP$^+$ cell counting (4 rats each; P=0.424 with 2-tailed Welch's t-test) and morphological parameters in dorsal hippocampus (4 rats each; intersections: two-way repeated-measures ANOVA for genotype×distance, $F_{6,36}$=0.259, P=0.952; genotype, $F_{1,6}$=0.014, P=0.910; distance, $F_{6,36}$ =602.3, P<1.00x10$^{-4}$; 0-60 μm P>0.05 with Bonferroni's; nodes: genotype×distance, $F_{6,36}$=1.50, P=0.205; genotype, $F_{1,6}$=1.72, P=0.238; distance, $F_{6,36}$=494.8, P<1.00x10$^{-4}$; 0-60 μm P>0.05 with Bonferroni's; dendritic length: genotype×radial distance $F_{6,36}$=1.11, P=0.373; genotype, $F_{1,6}$=1.07, P=0.341; distance, $F_{6,36}$=990.0, P<1.00 x 10$^{-4}$; 0-60 μm P>0.05 with Bonferroni's test; number of endings: genotype×radial distance $F_{6,36}$=1.00, P=0.440; genotype, $F_{1,6}$=2.28, P=0.181; distance, $F_{6,36}$=367.4, P<1.00x10$^{-4}$; 0-60 μm P>0.05 with Bonferroni's test). (**d**) Same as in **c** but for GFAP$^+$ cells in the pontine nuclei (4 rats per genotype; cell count: P=0.478 with 2-tailed Welch's t-test; intersections: two-way repeated-measures ANOVA for genotype×radial distance, $F_{6,36}$=0.85, P=0.538; genotype, $F_{1,6}$=2.78, P=0.147; distance,

$F_{6,36}$=437.6, P<1.00x10$^{-4}$; 0-60 μm P>0.05 with Bonferroni's; nodes: genotype×distance, $F_{6,36}$=0.313, P=0.927; genotype, $F_{1,6}$=0.158, P=0.705; distance, $F_{6,36}$=75.62, P<1.00 x 10$^{-4}$; 0-60 μm P>0.05 with Bonferroni's; dendritic length: genotype×distance $F_{6,36}$=0.858, P=0.535; genotype, $F_{1,6}$=0.571, P=0.478; distance, $F_{6,36}$=417.8, P<1.00x10$^{-4}$; 0-60 μm P>0.05 with Bonferroni's; endings: genotype×radial distance $F_{6,36}$=1.34, P=0.265; genotype, $F_{1,6}$=0.458, P=0.524; distance, $F_{6,36}$=148.9, P<1.00x10$^{-4}$; 0-60 μm P>0.05 with Bonferroni's). Source data are provided as a Source Data file.

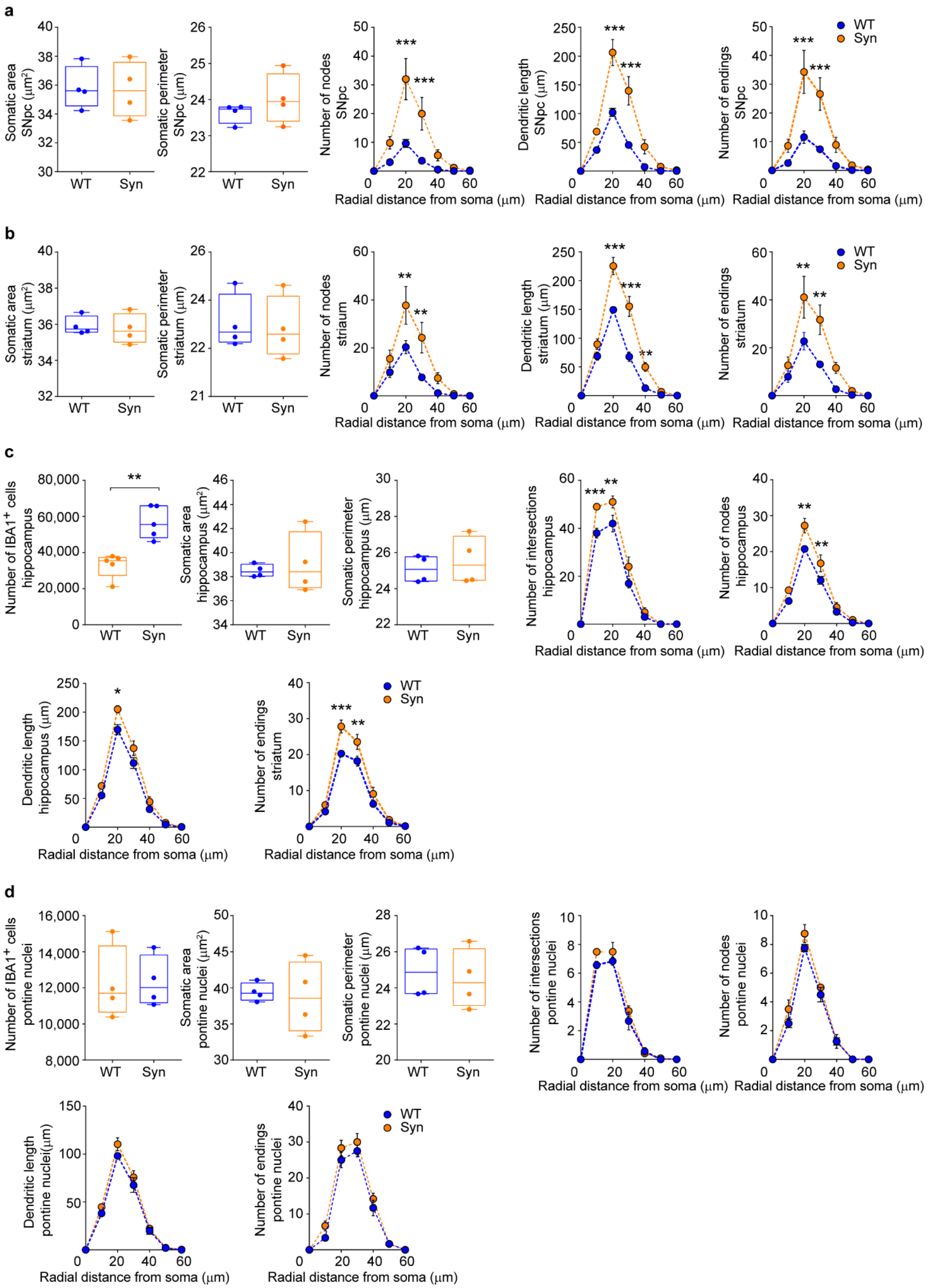
a
Somatic area SNpc (µm²)
Somatic perimeter SNpc (µm)
Number of nodes SNpc
Dendritic length SNpc (µm)
Number of endings SNpc
WT
Syn
Radial distance from soma (µm)
b
Somatic area striatum (µm²)
Somatic perimeter striatum (µm)
Number of nodes striaum
Dendritic length striatum (µm)
Number of endings striatum
c
Number of IBA1+ cells hippocampus
Somatic area hippocampus (µm²)
Somatic perimeter hippocampus (µm)
Number of intersections hippocampus
Number of nodes hippocampus
Dendritic length hippocampus (µm)
Number of endings striatum
d
Number of IBA1+ cells pontine nuclei
Somatic area pontine nuclei (µm²)
Somatic perimeter pontine nuclei (µm)
Number of intersections pontine nuclei
Number of nodes pontine nuclei
Dendritic length pontine nuclei(µm)
Number of endings pontine nuclei

**Supplementary Figure 4: Analysis of microglia cells in 4-month-old rats.** (**a**) Sholl analysis in SNpc (4 rats each; somatic area: $P>0.999$; somatic perimeter: $P=0.200$ with 2-tailed Mann-Whitney test; nodes: two-way repeated-measures ANOVA for genotype×distance, $F_{6,36}=8.76$, $P<1.00x10^{-4}$; genotype, $F_{1,6}=8.87$, $P=0.025$; distance, $F_{6,36}=27.74$, $P<1.00x10^{-4}$; 20 μm ***$P<1.00x10^{-4}$, 30 μm ***$P<5.00 \times 10^{-4}$, with Bonferroni's; dendritic length: genotype×distance $F_{6,36}=14.95$, $P<1.00x10^{-4}$; genotype, $F_{1,6}=16.22$, $P=0.007$; distance, $F_{6,36}=107.3$, $P<1.00x10^{-4}$; 20 μm ***$P<1.00x10^{-4}$, 30 μm ***$P<1.00x10^{-4}$ with Bonferroni's; number of endings: genotype×distance $F_{6,36}=9.03$, $P<1.0x10^{-4}$; genotype, $F_{1,6}=9.17$, $P=0.023$; distance, $F_{6,36}=34.83$, $P<1.00x10^{-4}$; 20 μm ***$P<1.00 \times 10^{-4}$, 30 μm ***$P=1.00x10^{-4}$, with Bonferroni's test). (**b**) As in (**a**) but for microglia in striatum (4 rats each; somatic area: $P=0.723$; somatic perimeter: $P=0.807$ with 2-tailed Welch's t-test; nodes: two-way repeated-measures ANOVA for genotype×distance, $F_{6,36}=4.82$, $P=0.001$; genotype, $F_{1,6}=4.83$, $P=0.070$; distance, $F_{6,36}=40.92$, $P<1.00x10^{-4}$; 20 μm **$P=0.002$, 30 μm **$P=0.003$, with Bonferroni's; dendritic length: genotype×distance, $F_{6,36}=17.88$, $P<1.00x10^{-4}$; genotype, $F_{1,6}=19.75$, $P=0.004$; distance, $F_{6,36}=274.0$, $P<1.00x10^{-4}$; 20 μm ***$P<1.00x10^{-4}$, 30 μm ***$P<1.00x10^{-4}$, 40 μm **$P=0.009$ with Bonferroni's; number of endings: genotype×distance, $F_{6,36}=4.73$, $P=0.001$; genotype, $F_{1,6}=5.64$, $P=0.055$; distance, $F_{6,36}=43.73$, $P<1.00 x10^{-4}$; 20 μm **$P=0.002$, 30 μm **$P=0.002$, with Bonferroni's). (**c**) $Iba^+$ cell counting in the dorsal hippocampus (5 rats per genotype; **$P=0.008$ with 2-tailed Mann-Whitney test) and morphological parameters (4 rats each; somatic area: $P=0.678$ with 2-tailed Welch's t-test; somatic perimeter: $P=0.565$ with 2-tailed Welch's t-test; intersections: two-way repeated measures ANOVA for genotype×distance, $F_{6,36}=4.13$, $P=0.003$; genotype, $F_{1,6}=8.01$, $P=0.030$; distance, $F_{6,36}=320.6$, $P<1.00x10^{-4}$; 10 μm ***$P=9.00x10^{-4}$, 20 μm **$P=0.009$, with Bonferroni's; nodes: genotype×distance, $F_{6,36}=5.02$, $P=8.00x10^{-4}$; genotype, $F_{1,6}=5.74$, $P=0.054$; distance, $F_{6,36}=258.3$, $P<1.00 \times 10^{-4}$; 20 μm ***$P=3.00 \times 10^{-4}$, 30 μm *$P=0.013$, with Bonferroni's; dendritic length: genotype×radial distance $F_{6,36}=2.17$, $P=0.069$; genotype, $F_{1,6}=6.86$, $P=0.040$; distance, $F_{6,36}=71.59$, $P<1.00x10^{-4}$; 20 μm *$P=0.043$ with Bonferroni's; number of endings:

genotype×radial distance $F_{6,36}$=6.14, P=2.00 x $10^{-4}$; genotype, $F_{1,6}$=6.60, P=0.042; distance, $F_{6,36}$= 298.9, P<1.00x$10^{-4}$; 20 μm ***P<1.00 x $10^{-4}$, 30 μm **P=0.006 with Bonferroni's). (**d**) Same as in (**c**) but for $Iba^{+}$ cells in pontine nuclei (4 rats each; cell count: P=0.931 with 2-tailed Welch's t-test; somatic area: P=0.806 with 2-tailed Welch's t-test; somatic perimeter: P=0.716 with 2-tailed Welch's t-test; intersections: two-way repeated measures ANOVA for genotype×distance, $F_{6,36}$=1.17, P=0.344; genotype, $F_{1,6}$=2.20, P=0.188; distance, $F_{6,36}$=278.5, P<1.00 x$10^{-4}$; WT versus Syn for 0-60 μm: P>0.05 with Bonferroni's; nodes: two-way repeated-measures ANOVA for genotype×distance from soma, $F_{6,36}$=1.25, P=0.303; genotype, $F_{1,6}$=2.14, P=0.194; distance, $F_{6,36}$=216.7, P<1.00x$10^{-4}$; WT versus Syn for 0-60 μm: P>0.05 with Bonferroni's ; dendritic length: two-way repeated-measures ANOVA for genotype×radial distance $F_{6,36}$=0.989, P= 0.448; genotype, $F_{1,6}$=2.04, P=0.204; distance, $F_{6,36}$=286.4, P<1.00x$10^{-4}$; WT versus Syn for 0-60 μm: P>0.05 with Bonferroni's test; number of endings: two-way repeated measures ANOVA for genotype × radial distance $F_{6,36}$ = 0.679, P = 0.667; genotype, $F_{1,6}$=3.38, P=0.116; distance, $F_{6,36}$=165.2, P<1.00x$10^{-4}$; WT versus Syn for 0-60 μm: P>0.05 with Bonferroni's test.

Source data are provided as a Source Data file.

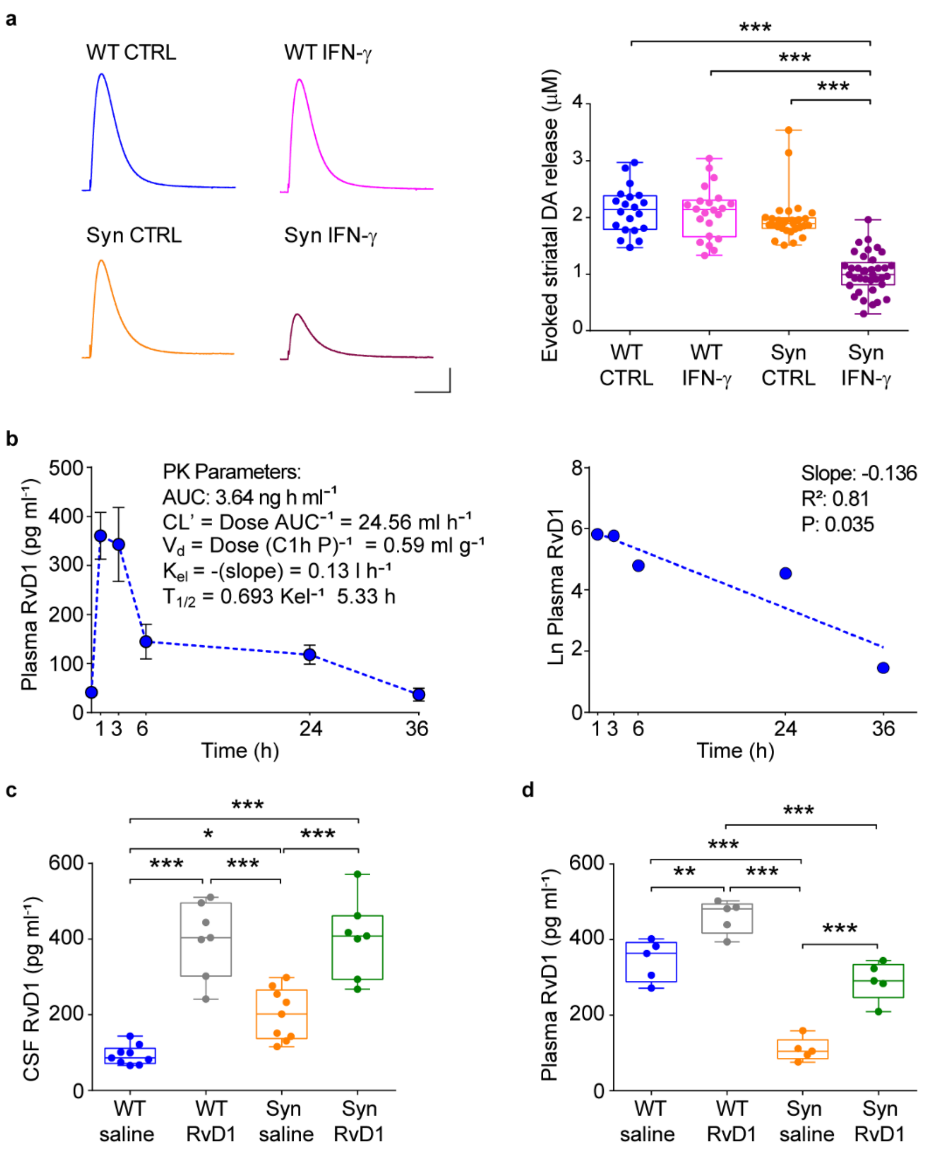


**Supplementary Figure 5: IFN-γ effects on nigrostriatal DA release in 2-month-old slices and additional data following RvD1 treatment.** (**a**) DA traces in slices of the dorsolateral striatum from 2-month-old rats, following incubation with IFN-γ (100-200 ng $ml^{-1}$) or aCSF for 3 h (scale: 500 ms, 50 pA). The plot shows decrease in evoked DA release in IFN-γ-treated slices from Syn rats compared with controls (IFN-γ: 22 slices from 4 WT, 37 slices from 4 Syn rats; CTRL: 20 slices from 4 WT, 29 slices from 4 Syn rats; two-way ANOVA: genotype×treatment, $F_{1,104}$=33.2, P<1.00x$10^{-4}$; genotype, $F_{1,104}$=59.33, P<1.00x$10^{-4}$; treatment, $F_{1,104}$=38.42, P<1.00x$10^{-4}$; WT/CTRL–Syn/IFN-γ

***$P<1.00 \times 10^{-4}$, WT/IFN-γ–Syn/IFN-γ ***$P<1.00 \times 10^{-4}$, Syn/CTRL–Syn/IFN-γ ***$P<1.00 \times 10^{-4}$, with Bonferroni's). (**b**) Time-course of RvD1 concentration in rat plasma collected after a single i.p. injection of RvD1 (0.2 μg $kg^{-1}$). Results are shown as mean ± s.e.m. from 5-7 rats per time point. The right plot shows the natural logarithmic (ln) of RvD1 plasma concentration after the maximum peak vs time, obtained by Pearson's correlation, which was used to calculate pharmacokinetics parameters: area under the curve (AUC), apparent clearance (CL'), apparent volume of distribution ($V_d$) normalized to the weight of RvD1-injected animals, $1^{st}$ order elimination rate ($K_{el}$) and elimination half-life ($T_{1/2}$). (**c,d**) Quantification with ELISA of RvD1 in the CSF (**c**) and plasma (**d**) of 4-month-old rats treated with saline or RvD1 (0.2 μg $kg^{-1}$, i.p.; twice a week for 2 months). RvD1 levels are increased in both CSF and plasma (**c**: 9 WT/saline, 7/WT RvD1 rats; 9 Syn/saline, 7 Syn/RvD1 rats; two-way ANOVA for genotype×treatment, $F_{1,28}=3.64$, $P=0.067$; genotype, $F_{1,28}=4.14$, $P=0.052$; treatment, $F_{1,28}=87.53$, $P<1.00 \times 10^{-4}$; WT/saline-WT/RvD1 ***$P<1.00 \times 10^{-4}$, WT/saline-Syn/saline *$P=0.035$, WT/saline-Syn/RvD1 ***$P<1.00 \times 10^{-4}$, WT/RvD1-Syn/saline ***$P=1.00 \times 10^{-4}$, Syn/saline-Syn/RvD1 ***$P<1.00 \times 10^{-4}$, with Bonferroni's; **d**: 5 rats each; two-way ANOVA for genotype×treatment, $F_{1,16}=2.53$, $P=0.131$; genotype, $F_{1,16}=97.06$, $P<1.00 \times 10^{-4}$; treatment, $F_{1,16}=51.84$, $P<1.00 \times 10^{-4}$; WT/saline-WT/RvD1 **$P=0.007$, WT/saline-Syn/saline ***$P<1.00 \times 10^{-4}$, WT/RvD1-Syn/saline ***$P<1.00 \times 10^{-4}$, WT/RvD1-Syn/RvD1 ***$P=2.00 \times 10^{-4}$, Syn/saline-Syn/RvD1 ***$P<1.00 \times 10^{-4}$, with Bonferroni's). Source data are provided as a Source Data file.

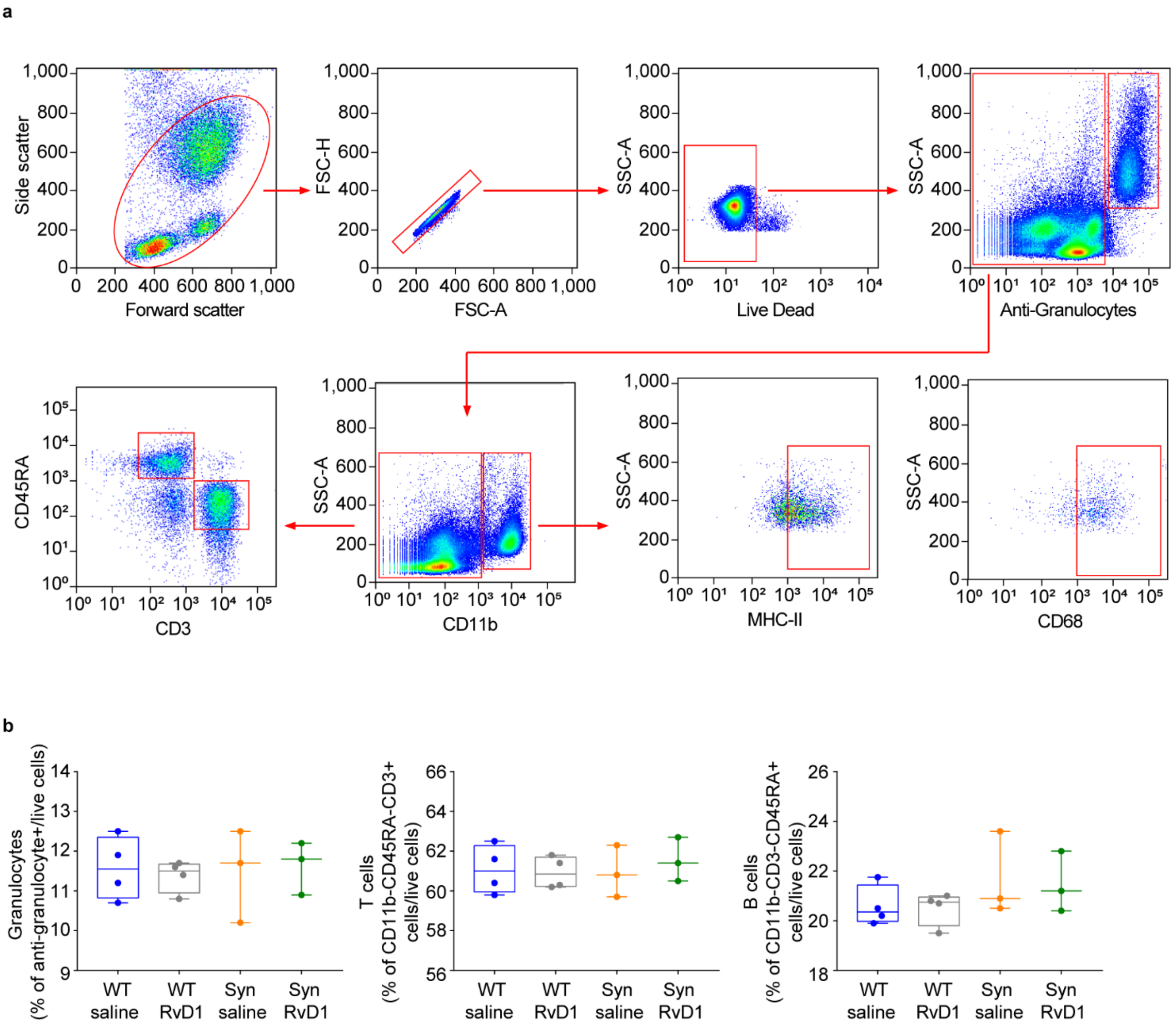


**Supplementary Figure 6: Polychromatic flow cytometry analysis of peripheral blood cells**. (**a**) Gating strategy for identification of the main cell populations of peripheral blood. Total leukocytes were gated and after excluding cell doublets and eventual dead cells, the % of cells expressing either anti-granulocytes or CD11b (monocytes) were plotted. Inside the $CD11b^-$ population, we plotted CD3 and CD45RA for the identification of T and B cells. $CD11b^+$ monocytes were further gated to observe the % expression of MHC-II and CD68. (**b**) Polychromatic flow cytometry results in 4-month-old rats following saline or RvD1 treatment, shown as % of each blood cell population (4 WT/saline, 4 WT/RvD1 rats; 3 Syn/saline, 3 Syn/RvD1 rats; granulocytes: two-way ANOVA for genotype×treatment, $F_{1,10}$=0.193, P=0.670; genotype, $F_{1,10}$=0.03, P=0.861; treatment, $F_{1,10}$=0.002,

P=0.969; T-lymphocytes: two-way ANOVA for genotype×treatment, $F_{1,10}$=0.40, P=0.543; genotype, $F_{1,10}$=0.15, P=0.703; treatment, $F_{1,10}$=0.14, P=0.713; B-lymphocytes: two-way ANOVA for genotype×treatment, $F_{1,10}$=0.01, P=0.926; genotype, $F_{1,10}$=2.98, P=0.115; treatment, $F_{1,10}$=0.06, P=0.813).

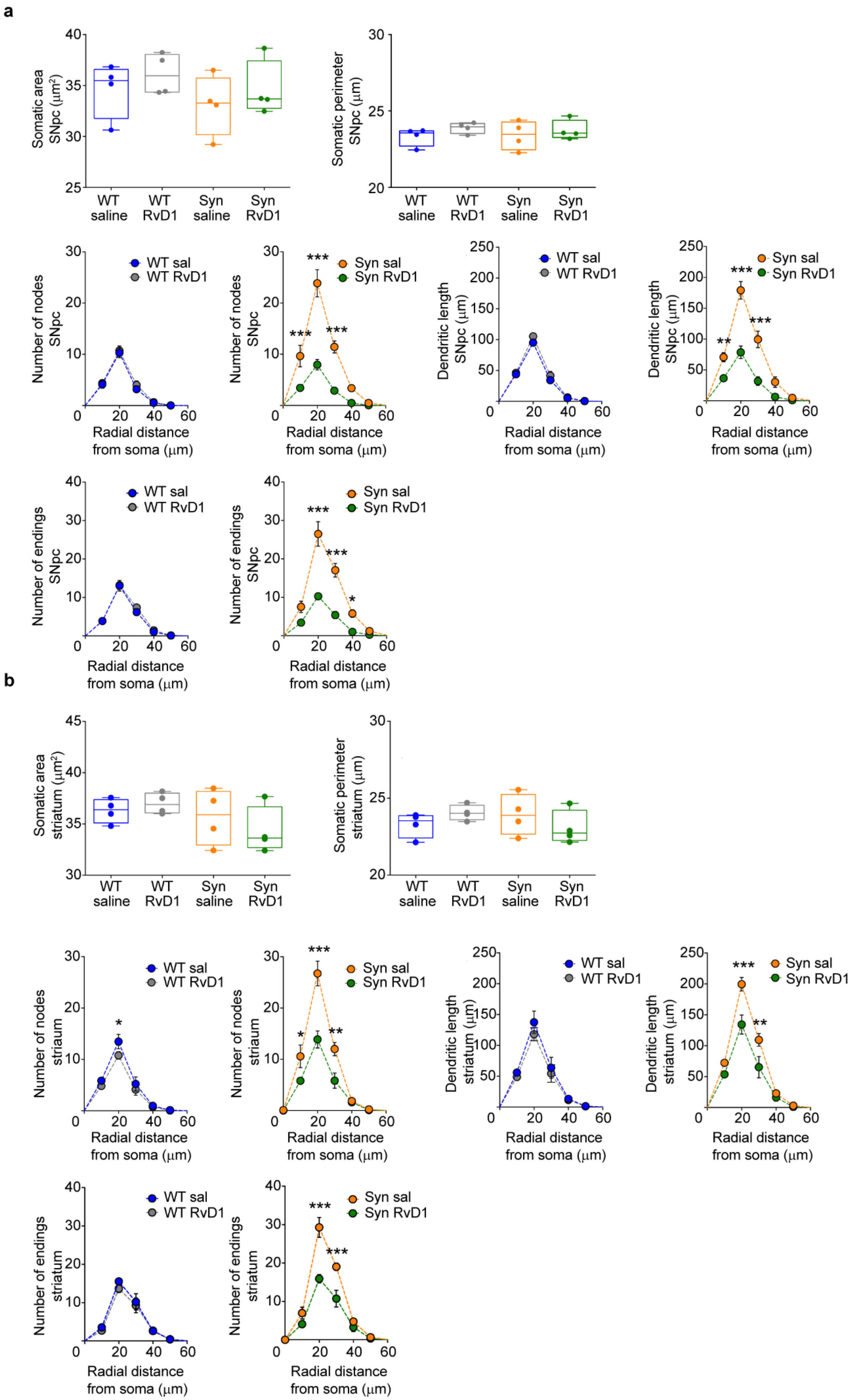
a
Somatic area SNpc (µm²)
Somatic perimeter SNpc (µm)
WT saline
WT RvD1
Syn saline
Syn RvD1
Number of nodes SNpc
Dendritic length SNpc (µm)
Number of endings SNpc
WT sal
WT RvD1
Syn sal
Syn RvD1
Radial distance from soma (µm)
b
Somatic area striatum (µm²)
Somatic perimeter striatum (µm)
Number of nodes striaum
Number of nodes striaum
Dendritic length striatum (µm)
Number of endings striatum
Radial distance from soma (µm)

**Supplementary Figure 7: Sholl analysis of microglia in the SNpc and striatum of 4-month-old treated rats.** (**a**) Iba$^+$ cell morphology parameters evaluated during Sholl analysis in the SNpc of 4-month-old treated rats (4 rats each; soma area: two-way ANOVA for genotype×treatment, $F_{1,12}$=3x10$^{-4}$, P=0.986; genotype, $F_{1,12}$=1.315, P=0.274; treatment, $F_{1,12}$=1.337, P=0.270; soma perimeter: two-way ANOVA for genotype×treatment, $F_{1,12}$=0.130, P=0.725; genotype, $F_{1,2}$=0.014, P=0.906; treatment, $F_{1,12}$=1.839, P=0.200; number of nodes WT: two-way repeated measures ANOVA for treatment×radial distance from soma, $F_{6,36}$=0.23, P=0.964; treatment, $F_{1,6}$=0.36, P=0.571; distance, $F_{6,36}$=159.6, P<1.00x10$^{-4}$; number of nodes Syn: two-way repeated measures ANOVA for treatment×radial distance, $F_{6,36}$=22.89, P<1.00 x 10$^{-4}$; treatment, $F_{1,6}$=28.84, P=0.002; distance, $F_{6,36}$=90.86, P<1.00x10$^{-4}$; 10 μm ***P=7.00x10$^{-4}$, 20 μm ***P<1.00x10$^{-4}$, 30 μm ***P<1.00x10$^{-4}$, with Bonferroni's; dendritic length WT: two-way repeated measures ANOVA for treatment×radial distance, $F_{6,36}$=0.95, P=0.475; treatment, $F_{1,6}$=0.69, P=0.241; distance, $F_{6,36}$=319, P<1.00x10$^{-4}$; dendritic length Syn: two-way repeated measures ANOVA for treatment×radial distance, $F_{6,36}$=24.71, P<1.00 x 10$^{-4}$; treatment, $F_{1,6}$=22.87, P=0.003; distance, $F_{6,36}$=154, P<1.00x10$^{-4}$; 10 μm **P=0.009, 20 μm ***P<1.00x10$^{-4}$, 30 μm ***P<1.00 x 10$^{-4}$, with Bonferroni's; number of endings WT: two-way repeated measures ANOVA for treatment×radial distance, $F_{6,36}$=0.376, P=0.889; treatment, $F_{1,6}$=0.30, P=0.603; distance, $F_{6,36}$=185.5, P<1.00x10$^{-4}$; number of endings Syn: two-way repeated measures ANOVA for treatment×radial distance, $F_{6,36}$=18.48, P<1.00x10$^{-4}$; treatment, $F_{1,6}$=32.78, P=0.001; distance, $F_{6,36}$=89.5, P<1.00x10$^{-4}$; 20 μm ***P<1.00x10$^{-4}$, 30 μm ***P<1.00x10$^{-4}$, 40 μm *P=0.042, with Bonferroni's test). (**b**) As in (**a**), but for microglia in the striatum (4 rats each; soma area: two-way ANOVA for genotype×treatment, $F_{1,12}$=1.108, P=0.313; genotype, $F_{1,12}$=2.828, P=0.119; treatment, $F_{1,12}$=0.106, P=0.750; soma perimeter: two-way ANOVA for genotype×treatment, $F_{1,12}$=2.766, P=0.122; genotype, $F_{1,12}$=0.109, P=0.747; treatment, $F_{1,12}$=0.007, P=0.937; number of nodes WT: two-way repeated measures ANOVA for treatment×radial distance from soma, $F_{6,36}$=1.45, P=0.224; treatment, $F_{1,6}$=1.846, P=0.223; distance, $F_{6,36}$=121.2, P<1.00x10$^{-4}$;

20 μm *P=0.040, with Bonferroni's post hoc test; number of nodes Syn rat: two-way repeated measures ANOVA for treatment×radial distance from soma, $F_{6,36}$=11.7, $P<1.00x10^{-4}$; treatment, $F_{1,6}$=13.87, P=0.009; distance, $F_{6,36}$=112.3, $P<1.00x10^{-4}$; 10 μm *P=0.035, 20 μm ***$P<1.00x10^{-4}$, 30 μm **P=0.003, with Bonferroni's test; dendritic length WT: two-way repeated measures ANOVA for treatment×radial distance, $F_{6,36}$=0.505, P=0.800; treatment, $F_{1,6}$=0.55, P=0.484; distance, $F_{6,36}$=88.65, $P<1.00x10^{-4}$; dendritic length Syn: two-way repeated measures ANOVA for treatment×distance, $F_{6,36}$=6.96, $P<1.00x10^{-4}$; treatment, $F_{1,6}$=9.93, P=0.020; distance, $F_{6,36}$=166.5, $P< 1.00x10^{-4}$; 20 μm ***$P<1.00x10^{-4}$, 30 μm **P=0.002, with Bonferroni's test; number of endings WT: two-way repeated measures ANOVA for treatment×radial distance, $F_{6,36}$=0.402, P=0.873; treatment, $F_{1,6}$=0.446, P=0.529; distance, $F_{6,36}$=93.21, $P<1.00x10^{-4}$; number of endings Syn: two-way repeated measures ANOVA for treatment×radial distance, $F_{6,36}$=12.56, $P<1.00 \text{ x } 10^{-4}$; treatment, $F_{1,6}$=17.9, P=0.006; distance, $F_{6,36}$=144, $P<1.00x10^{-4}$; 20 μm ***$P<1.00x10^{-4}$, 30 μm ***$P<1.00x10^{-4}$, with Bonferroni's test).

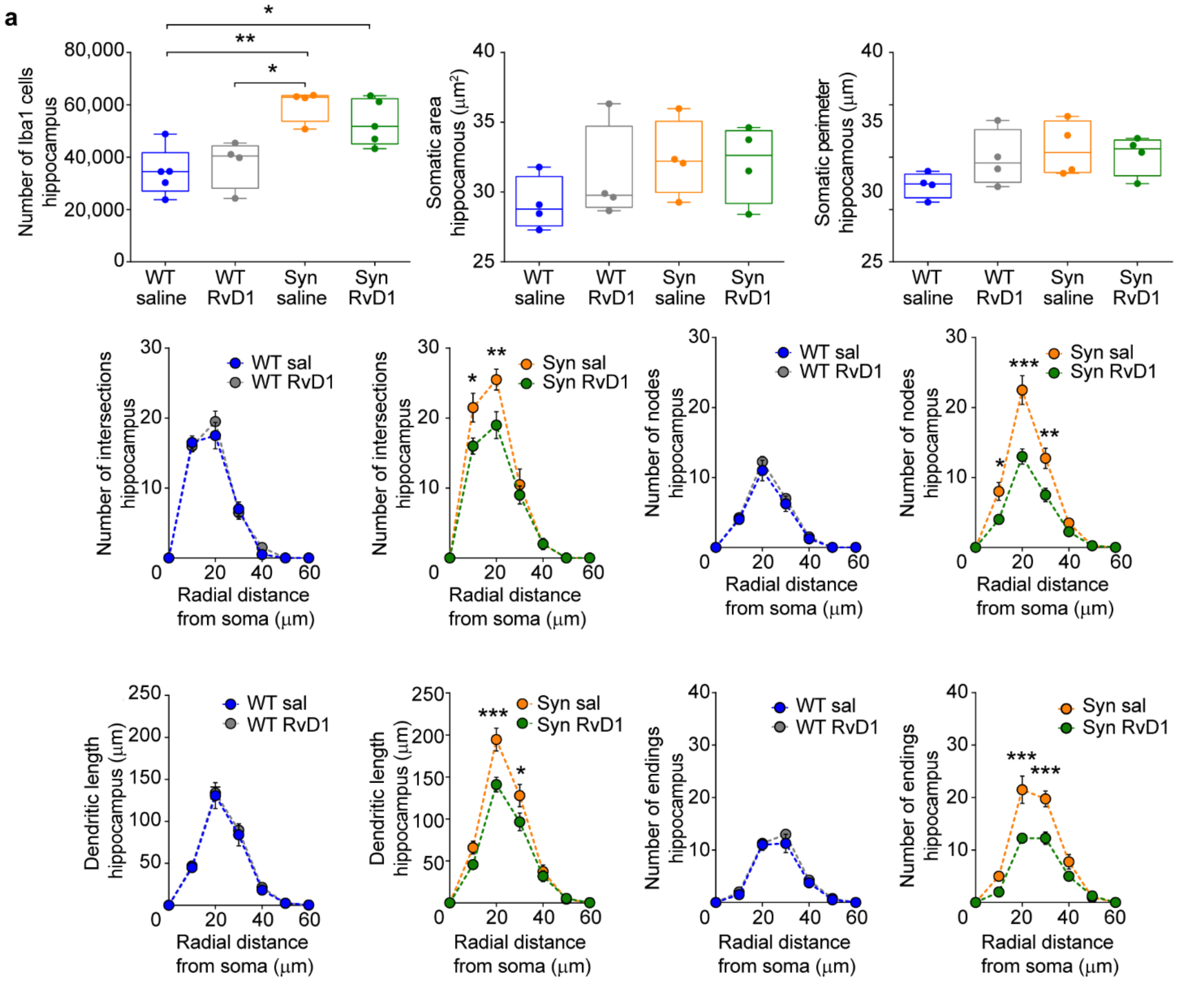


**Supplementary Figure 8: Microglia analysis in the dorsal hippocampus of 4-month-old treated rats.** (**a**) Iba$^{+}$ cell count in the dorsal hippocampus of 4-month-old treated rats (5 WT/saline, 4 WT/RvD1, 4 Syn/saline, 5 Syn/RvD1; two-way ANOVA for genotype×treatment, $F_{1,14}$=1.52, P=0.238; genotype, $F_{1,14}$=26.11, P=2.00x10$^{-4}$; treatment, $F_{1,14}$=0.19, P=0.673; WT/saline-Syn/saline **P=0.003, WT/saline-Syn/RvD1 *P=0.021, WT/RvD1-Syn/saline *P=0.014, with Bonferroni's test) and morphology parameters evaluated during Sholl analysis (4 rats each; soma area: two-way ANOVA for genotype×treatment, $F_{1,12}$=0.691, P=0.422; genotype, $F_{1,12}$=2.27, P=0.158; treatment, $F_{1,12}$=0.339, P=0.571; soma perimeter: two-way ANOVA for genotype×treatment, $F_{1,12}$=2.11, P=0.172; genotype, $F_{1,2}$=3.04, P=0.107; treatment, $F_{1,12}$=0.829, P=0.381; number of intersections WT: two-way repeated measures ANOVA for treatment×radial distance from soma, $F_{6,36}$=0.74,

$P=0.618$; treatment, $F_{1,6}=0.18$, $P=0.688$; distance, $F_{6,36}=238.6$, $P<1.00 \times 10^{-4}$; number of intersections Syn: two-way repeated measures ANOVA for treatment×radial distance, $F_{6,36}=3.69$, $P=0.006$; treatment, $F_{1,6}=4.18$, $P=0.087$; distance, $F_{6,36}=166.2$, $P<1.00\times10^{-4}$; 10 μm *$P=0.014$, 20 μm **$P=0.002$, with Bonferroni's; number of nodes WT: two-way repeated measures ANOVA for treatment×radial distance, $F_{6,36}=0.45$, $P=0.837$; treatment, $F_{1,6}=0.59$, $P=0.471$; distance, $F_{6,36}=157.1$, $P<1.00\times10^{-4}$; number of nodes Syn: two-way repeated measures ANOVA for treatment×radial distance, $F_{6,36}=10.5$, $P<1.00\times10^{-4}$; treatment, $F_{1,6}=14.12$, $P=0.009$; distance, $F_{6,36}=143$, $P<1.00\times10^{-4}$; 10 μm *$P=0.023$, 20 μm ***$P<1.00\times10^{-4}$, 30 μm **$P=0.001$, with Bonferroni's; dendritic length WT: two-way repeated measures ANOVA for treatment×radial distance, $F_{6,36}=0.095$, $P=0.997$; treatment, $F_{1,6}=0.12$, $P=0.744$; distance, $F_{6,36}=209.2$, $P<1.00\times10^{-4}$; dendritic length Syn: two-way repeated measures ANOVA for treatment×radial distance, $F_{6,36}=5.65$, $P=3.00\times10^{-4}$; treatment, $F_{1,6}=6.31$, $P=0.046$; distance, $F_{6,36}=219.6$, $P<1.00\times10^{-4}$; 20 μm ***$P<1.00\times10^{-4}$, 30 μm *$P=0.026$, with Bonferroni's; number of endings WT: two-way repeated-measures ANOVA for treatment×radial distance, $F_{6,36}=0.628$, $P=0.707$; treatment, $F_{1,6}=0.64$, $P=0.453$; distance, $F_{6,36}=190.0$, $P<1.00\times10^{-4}$; number of endings Syn: two-way repeated measures ANOVA for treatment×radial distance, $F_{6,36}=9.07$, $P<1.00\times10^{-4}$; treatment, $F_{1,6}=13.99$, $P=0.009$; distance, $F_{6,36}=132.3$, $P<1.00\times10^{-4}$; 20 μm ***$P<1.00\times10^{-4}$, 30 μm ***$P<1.00\times10^{-4}$, with Bonferroni's). Source data are provided as a Source Data file.

**Supplementary Table 1: Clinical profile of the 8 PD patients used in the study**

| | **PD-1** | **PD-2** | **PD-3** | **PD-4** | **PD-5** | **PD-6** | **PD-7** | **PD-8** |
|---|---|---|---|---|---|---|---|---|
| **Sex** | M | F | M | F | M | F | F | M |
| **Age** | 52 | 68 | 67 | 54 | 73 | 53 | 69 | 65 |
| **Comorbidity** | none | none | none | none | none | none | depression | none |
| **Symptom duration (months)** | 8 | 12 | 12 | 8 | 7 | 10 | 6 | 10 |
| **MMSE score** | 30 | 27 | 27 | 28 | 26 | 29 | 29 | 28 |
| **H&Y scale** | 1 | 2 | 2 | 1 | 1 | 1 | 1 | 2 |
| **UPDRS III total score** | 12 | 30 | 29 | 19 | 25 | 22 | 19 | 25 |
| Speech | 0 | 1 | 1 | 0 | 1 | 0 | 2 | 1 |
| Facial expression | 2 | 2 | 1 | 1 | 2 | 2 | 2 | 1 |
| Rest tremor upper limb (R) | 0 | 0 | 0 | 0 | 0 | 1 | 0 | 0 |
| Rest tremor upper limb (L) | 0 | 0 | 0 | 0 | 2 | 1 | 0 | 0 |
| Rest tremor lower limb (R) | 0 | 0 | 0 | 0 | 0 | 0 | 0 | 0 |
| Rest tremor lower limb (L) | 0 | 0 | 0 | 0 | 0 | 0 | 0 | 0 |
| Action/posture tremor upper limb (R) | 0 | 0 | 1 | 3 | 0 | 0 | 0 | 0 |
| Action/posture tremor upper limb (L) | 1 | 0 | 0 | 0 | 0 | 0 | 0 | 0 |
| Neck rigidity | 1 | 2 | 1 | 0 | 2 | 1 | 3 | 2 |
| Rigidity upper limb (R) | 0 | 2 | 2 | 2 | 1 | 2 | 1 | 2 |
| Rigidity upper limb (L) | 1 | 2 | 1 | 1 | 2 | 1 | 2 | 1 |
| Rigidity lower limb (R) | 0 | 1 | 1 | 0 | 1 | 0 | 0 | 1 |
| Rigidity lower limb (L) | 0 | 1 | 1 | 0 | 1 | 0 | 0 | 1 |
| Finger tapping (R) | 1 | 2 | 3 | 2 | 1 | 2 | 2 | 2 |
| Finger tapping (L) | 2 | 2 | 2 | 2 | 2 | 1 | 1 | 1 |
| Hand gripping (R) | 0 | 2 | 2 | 1 | 1 | 2 | 1 | 1 |
| Hand gripping (L) | 1 | 2 | 1 | 2 | 2 | 1 | 1 | 1 |
| Rapidly alternating hand movements (R) | 0 | 2 | 3 | 1 | 0 | 2 | 1 | 2 |
| Rapidly alternating hand movements (L) | 1 | 2 | 2 | 1 | 2 | 1 | 1 | 1 |
| Foot stepping (R) | 0 | 1 | 1 | 1 | 1 | 1 | 1 | 2 |
| Foot stepping (L) | 1 | 1 | 0 | 1 | 1 | 0 | 0 | 1 |
| Arising from chair | 0 | 1 | 1 | 0 | 0 | 0 | 0 | 1 |
| Posture | 0 | 1 | 2 | 0 | 1 | 1 | 0 | 1 |
| Gait | 0 | 0 | 0 | 0 | 0 | 1 | 0 | 0 |
| Postural stability | 0 | 1 | 1 | 0 | 0 | 0 | 0 | 1 |
| Bradykinesia/hypokinesia | 1 | 2 | 2 | 1 | 2 | 2 | 1 | 2 |

Scoring was done using the UPDRS rating scale: 0, normal or no problems; 1, minimal problems; 2, mild problems; 3, moderate problems; 4, severe problems (R: right; L: left)
