# Supplementary material for: Blunting neuroinflammation with resolvin D1 prevents early pathology in a rat model of Parkinson’s disease
Source: Nat Commun. 2019 Sep 2;10:3945. doi: 10.1038/s41467-019-11928-w (PMC6718379; doi:10.1038/s41467-019-11928-w)
Supplement: Supplementary file 3 — Reporting Summary [file 41467_2019_11928_MOESM3_ESM.pdf]

nature research

Corresponding author(s): Nicola Biagio Mercuri

Reporting Summary

Nature Research wishes to improve the reproducibility of the work that we publish. This form provides structure for consistency and transparency in reporting. For further information on Nature Research policies, see Authors & Referees and the Editorial Policy Checklist.

Statistical parameters

When statistical analyses are reported, confirm that the following items are present in the relevant location (e.g. figure legend, table legend, main text, or Methods section).

n/a	Confirmed	
☐	☒	The exact sample size (n) for each experimental group/condition, given as a discrete number and unit of measurement
☐	☒	An indication of whether measurements were taken from distinct samples or whether the same sample was measured repeatedly
☐	☒	The statistical test(s) used AND whether they are one- or two-sided<br>*Only common tests should be described solely by name; describe more complex techniques in the Methods section.*
☐	☒	A description of all covariates tested
☐	☒	A description of any assumptions or corrections, such as tests of normality and adjustment for multiple comparisons
☐	☒	A full description of the statistics including central tendency (e.g. means) or other basic estimates (e.g. regression coefficient) AND variation (e.g. standard deviation) or associated estimates of uncertainty (e.g. confidence intervals)
☐	☒	For null hypothesis testing, the test statistic (e.g. F, t, r) with confidence intervals, effect sizes, degrees of freedom and P value noted<br>*Give P values as exact values whenever suitable.*
☒	☐	For Bayesian analysis, information on the choice of priors and Markov chain Monte Carlo settings
☒	☐	For hierarchical and complex designs, identification of the appropriate level for tests and full reporting of outcomes
☒	☐	Estimates of effect sizes (e.g. Cohen's d, Pearson's r), indicating how they were calculated
☐	☒	Clearly defined error bars<br>*State explicitly what error bars represent (e.g. SD, SE, CI)*

Our web collection on statistics for biologists may be useful.

Software and code

Policy information about availability of computer code

Data collection	Behavioral experiments: Smart PanLab V3.0 (Harvard Apparatus); Electrophysiology: pClamp V9.0 or 10.0 (Molecular Devices); Microfluorometry: Till Photonics V4.0; Sholl analysis: Neurolucida 7.5 software (MicroBright-Field) ; cell count: Stereo Investigator System (MicroBrightField).
Data analysis	ImageJ for flurescence intensity; pClamp 10.0 for electrophysiology; Prism V7.0 for statistics

For manuscripts utilizing custom algorithms or software that are central to the research but not yet described in published literature, software must be made available to editors/reviewers upon request. We strongly encourage code deposition in a community repository (e.g. GitHub). See the Nature Research guidelines for submitting code & software for further information.

Data

Policy information about availability of data

All manuscripts must include a data availability statement. This statement should provide the following information, where applicable:

- Accession codes, unique identifiers, or web links for publicly available datasets
- A list of figures that have associated raw data
- A description of any restrictions on data availability

All figures (main and supplementary) contain raw data. The datasets generated and analysed during the study are available in the Source Data file accompanying the paper or can be obtained from the corresponding author on request.

Field-specific reporting

Please select the best fit for your research. If you are not sure, read the appropriate sections before making your selection.

☒ Life sciences ☐ Behavioural & social sciences ☐ Ecological, evolutionary & environmental sciences

For a reference copy of the document with all sections, see nature.com/authors/policies/ReportingSummary-flat.pdf

Life sciences study design

All studies must disclose on these points even when the disclosure is negative.

Sample size	The number of samples in each group and for each experiment was determined based on published studies. Number of animals was determined by power analysis based on the obtained values of sd and delta.
Data exclusions	no data were excluded from the analysis
Replication	For ELISA: samples were loaded twice, reported values are the means of the two measures. For other experiments (electrophysiology, cell count and morphology, cytometry, immunofluorescence, behavior), reproducibility was confirmed by repeating experiments in more animals per group.
Randomization	All randomization was performed by assigning a random number to each animal and using a random number table
Blinding	All data were collected by researchers blind to the genotype or pharmacological treatment of each animal.

Reporting for specific materials, systems and methods

Materials & experimental systems

n/a	Involved in the study	
☒	☐	Unique biological materials
☐	☒	Antibodies
☒	☐	Eukaryotic cell lines
☒	☐	Palaeontology
☐	☒	Animals and other organisms
☐	☒	Human research participants

Methods

n/a	Involved in the study	
☒	☐	ChIP-seq
☐	☒	Flow cytometry
☒	☐	MRI-based neuroimaging

Antibodies

Antibodies used	Primary antibodies for immunofluorescence: TH (1:700, Millipore; MAB318; RRID: AB_2201528), GFAP (1:200, DAKO, Z0334; RRID: AB_2314535), Iba1 (1:400, Wako #019-19741; RRID: AB_839504).<br><br>For flow cytometry: anti-granulocytes-FITC (REA535, Miltenyi Biotec; RRID: AB_2651885), CD3-PE-Vio770 (REA223, Miltenyi Biotec; RRID: AB_2657102), CD45RA-PE (OX-33, Biolegend; RRID: AB_314010), CD11b/c-APC (clone REA325, Miltenyi Biotec; RRID:AB_2752041), MHC-II-VioBlue (REA510, Miltenyi Biotec; RRID:AB_2652891), CD68-APC-Vio770 (REA237, Miltenyi Biotec; RRID:AB_2659019).<br><br>For Dot blot: anti-Alpha-synuclein filament antibody [MJFR-14-6-4-2] (Abcam, ab209538; RRID:AB_2714215), anti-Actin (1:60,000, Sigma-Aldrich, A5060; RRID:AB_476738)

	Secondary antibodies: Alexa Fluor 488 donkey anti-mouse IgG (1:200; RRID: AB_2556542 and Alexa Fluor 555 donkey anti-rabbit IgG (1:200; RRID: AB_162543).
Validation	All primary antibodies have specificity for rat, and can be applied for immunofluorescence , immunohistochemistry or MACS flow cytometry experiments, according to the manufacturers' websites.

Animals and other organisms

Policy information about studies involving animals; ARRIVE guidelines recommended for reporting animal research

Laboratory animals	Male homozygous BAC transgenic rats (Sprague-Dawley background) over-expressing the full-length human SNCA locus under the control of the endogenous human regulatory elements (Syn rats) and WT Sprague-Dawley were used at 2, 4, 18 months of age
Wild animals	the study did not involve wild animals
Field-collected samples	the study did not involve field-collected samples

Human research participants

Policy information about studies involving human research participants

Population characteristics	8 Parkinson's patients (4 male, 4 female) and 8 age-matched healthy controls (3 male, 5 female)
Recruitment	Recruitment was performed at the Neurology Clinic of the Tor Vergata University Hospital. PD was diagnosed according to the British Parkinson's Disease Society Brain Bank (UK-PDSBB) criteria. All PD patients were at early disease stage and were also untreated ("de novo", not taking levodopa, monoamine oxidase inhibitors or DA receptor agonists). The control group included age-matched subjects without degenerative and inflammatory diseases, not presenting motor or cognitive disturbances (e.g. patients with psychogenic disorders). Exclusion criteria for this study were: age younger than 50 or older than 80, dementia (Mini-Mental State Examination, MMSE score < 24), treatment with anti-inflammatory drugs in the last month, history of autoimmune/inflammatory diseases, cancer, thyroid disorders, diabetes or any other acute condition. PD patients were further evaluated with the Unified Parkinson's Disease Rating Scale (UPDRS) part III and Hoehn and Yahr (H&Y) scale for motor signs.

Flow Cytometry

Plots

Confirm that:

☒ The axis labels state the marker and fluorochrome used (e.g. CD4-FITC).

☒ The axis scales are clearly visible. Include numbers along axes only for bottom left plot of group (a 'group' is an analysis of identical markers).

☒ All plots are contour plots with outliers or pseudocolor plots.

☒ A numerical value for number of cells or percentage (with statistics) is provided.

Methodology

Sample preparation	Peripheral blood of rats was collected, lysed with 1X red blood cell lysis buffer for 10 min at room temperature and then stained for 30 min at 4°C with the different fluorochrome-conjugated antibodies.
Instrument	Cytoflex (Beckman Coulter)
Software	Flowjo V8
Cell population abundance	No cell sorting or cell purification is involved in this study but an immunophenotyping of the different cell populations of blood. In order to do so, at least 300,000 events were acquired, so that in the subsequent gatings enough cells were clearly visible.
Gating strategy	Total leukocytes were gated and after excluding both cell doublets and eventual dead cells (using LIVE DEAD Zombie Aqua dye), the % of cells expressing either anti-granulocytes and CD11b (monocytes) were plotted. Inside the CD11b- population, we plotted CD3 and CD45RA for the identification of T and B cells. CD11b+ monocytes were further gated to observe the % expression of MHC-II and CD68.

☒ Tick this box to confirm that a figure exemplifying the gating strategy is provided in the Supplementary Information.
